# Predicting the Functional Impact of *CDH1* Missense Mutations in Hereditary Diffuse Gastric Cancer

**DOI:** 10.3390/ijms18122687

**Published:** 2017-12-12

**Authors:** Soraia Melo, Joana Figueiredo, Maria Sofia Fernandes, Margarida Gonçalves, Eurico Morais-de-Sá, João Miguel Sanches, Raquel Seruca

**Affiliations:** 1 Instituto de Investigação e Inovação em Saúde (i3S), University of Porto, 4200-135 Porto, Portugal; soraiam@ipatimup.pt (S.M.); jfigueiredo@ipatimup.pt (J.F.); sfernandes@ipatimup.pt (M.S.F.); m.goncalves@i3s.up.pt (M.G.); eurico.sa@ibmc.up.pt (E.M.-d.-S.); 2 Institute of Molecular Pathology and Immunology, University of Porto (IPATIMUP), 4200-135 Porto, Portugal; 3 Medical Faculty, University of Porto, 4200-135 Porto, Portugal; 4 Institute for Systems and Robotics (ISR), Instituto Superior Técnico (IST), 1049-001 Lisboa, Portugal; jmrs@tecnico.ulisboa.pt; 5 Instituto de Biologia Molecular e Celular (IBMC), Universidade do Porto, 4200-135 Porto, Portugal

**Keywords:** Hereditary Diffuse Gastric Cancer, E-cadherin, *CDH1* missense variants, functional characterization, diagnostic tools

## Abstract

The role of E-cadherin in Hereditary Diffuse Gastric Cancer (HDGC) is unequivocal. Germline alterations in its encoding gene (*CDH1*) are causative of HDGC and occur in about 40% of patients. Importantly, while in most cases *CDH1* alterations result in the complete loss of E-cadherin associated with a well-established clinical impact, in about 20% of cases the mutations are of the missense type. The latter are of particular concern in terms of genetic counselling and clinical management, as the effect of the sequence variants in E-cadherin function is not predictable. If a deleterious variant is identified, prophylactic surgery could be recommended. Therefore, over the last few years, intensive research has focused on evaluating the functional consequences of *CDH1* missense variants and in assessing E-cadherin pathogenicity. In that context, our group has contributed to better characterize *CDH1* germline missense variants and is now considered a worldwide reference centre. In this review, we highlight the state of the art methodologies to categorize *CDH1* variants, as neutral or deleterious. This information is subsequently integrated with clinical data for genetic counseling and management of *CDH1* variant carriers.

## 1. Introduction

In this review article, a special focus is given to a particular type of gastric cancer, the Hereditary Diffuse Gastric Cancer (HDGC). Herein, important aspects of HDGC are discussed, including the molecular mechanisms involved, how E-cadherin deregulation affects the development of the disease, and more importantly the translation of this knowledge into clinical practice.

An overview is given of the role of E-cadherin in normal epithelia and cancer, how distinct missense mutations in the E-cadherin encoding gene, *CDH1*, differently disturb E-cadherin expression and function, what are the recommendations and guidelines for the classification and management of *CDH1* mutation carriers, and what strategies are available, or being developed, to predict *CDH1* variants pathogenicity. The latter includes in silico tools, in vitro assays for the analysis of E-cadherin expression profiles, intracellular organization, cell-cell adhesion status and invasive and migratory properties, and finally an in vivo approach taking advantage of the fly *Drosophila melanogaster*. We describe the technological developments and state of the art methodologies that have emerged, and how bench results are used to help clinicians and genetic counselors in the management of HDGC patients and families. In order to collect the available literature, related to germline E-cadherin missense mutations in the HDGC context, the PubMed database was accessed and publications searched from November 1982 to September 2017. Search terms included: *CDH1*, E-cadherin, E-cadherin in cancer, gastric cancer, familial and Hereditary Diffuse Gastric Cancer, E-cadherin dysfunction, E-cadherin germline mutation, *CDH1*/E-cadherin missense mutation, and in vitro and in vivo functional assays. Overall, this review brings together issues that are of interest to researchers, clinicians, and genetic counseling experts.

## 2. The Role of E-Cadherin in Normal Epithelia and Cancer

Cell-cell adhesion is critical for the maintenance of tissue morphogenesis and homeostasis, but is also crucial for a plethora of other cellular processes, including cell differentiation, survival, and migration through the control of gene expression and the activation of signaling pathways [1]. Particularly relevant in cell-cell adhesion are the classical cadherins, such as E-cadherin, that play a key role in calcium-dependent cell-cell interactions, in establishing tight adherent junctions, and in defining cell differentiation specificity [2]. In fact, the cytoplasmic tail of E-cadherin forms a protein complex with β-, p120- and α-catenins that links this adhesion molecule with the actin-myosin network, coordinating the shape, polarity, and function of the cells in an epithelium [3,4]. Given the broad-ranging functions of E-cadherin on tissue organization, it is not surprising that alterations in its expression or structural modifications in its encoding gene *CDH1* are common events during cancer progression and contribute to the aberrant morphogenetic effects in cancer [3,5,6]. Indeed, most human carcinomas partially or completely lose E-cadherin as they progress towards malignancy, supporting the role of E-cadherin and downstream targets in cancer development [3,7].

## 3. E-Cadherin Deregulation Mechanisms 

Mutations in the *CDH1* gene are a well-known mechanism of E-cadherin deregulation, as thoroughly described in Section 4. In addition, downregulation of E-cadherin expression can occur via other mechanisms including overexpression of transcription repressors, alterations of microRNAs (miRNAs), deregulation of protein trafficking, and aberrant post-translational regulation of the protein [7,8,9]. The transcriptional activity of E-cadherin can be negatively regulated by a multitude of transcriptional repressors like SNAIL, with expression levels increased in ductal breast carcinomas [10], but also Slug, zinc finger E-box-binding homeobox 1 (ZEB1), and ZEB2 [11,12]. Inhibition of members of miR-200 family of miRNAs, which directly target the transcriptional repressors of E-cadherin (ZEB1 and ZEB2), was shown to induce the reduction of E-cadherin mRNA levels, and miR-9 and miR-101 have also been implicated in the complex network of E-cadherin regulation [13]. Further, deregulation of exocytic and endocytic pathways is known to control the delivery and internalization of E-cadherin, with consequences for protein turnover, recycling, sequestration, and degradation [14]. In particular, the disruption of the binding of type Iγ phosphatidylinositol phosphate kinase (PIPKIγ) to E-cadherin modulates the intracellular trafficking, inducing aberrant E-cadherin transport and blocking the gathering of the adherent junctions [15]. Another key molecule in the endocytic pathway is the ADP-ribosylation factor 6 (ARF6) [16,17], whose activation through epithelial growth factor receptor (EGFR) signaling induces E-cadherin internalization into early endosomes [18]. In fact, abnormal activation of proto-oncogenes such as EGFR, c-Met, and Src also results in increased phosphorylation of tyrosine residues in the E-cadherin-catenin complex [7], which leads to internalization and ubiquitination of the protein through the recruitment of E3-ubiquitin ligase Hakai [19]. More recently, post-translational glycosylation of E-cadherin has also been suggested as a mechanism of deregulation in many pathophysiological steps of tumour development and progression [20]. More specifically, E-cadherin extracellular domain has four potential *N*-glycosylation sites essential for its correct folding and transport to the cell membrane [20]. The abrogation of one of those specific residues (Asn633) was demonstrated to target E-cadherin for endoplasmic reticulum-associated degradation (ERAD) [21]. At the cytoplasmic region, E-cadherin undergoes *O*-glycosylation (*O*-GlcNAc) that blocks the transport of newly synthesised molecules to the cell surface and prevents the process of intercellular adhesion via p120-catenin [22].

## 4. The Hereditary Diffuse Gastric Cancer and Its Genetic Signature 

The deregulation of E-cadherin is particularly well established in gastric cancer. More specifically, in the diffuse type of gastric cancer, E-cadherin somatic mutations were described in up to 40–70% of the cases. Moreover, germline loss-of-function mutations are the only proven cause of the cancer syndrome HDGC, occurring in approximately 40% of cases [23,24,25]. In fact, the first evidence of an inherited form of diffuse gastric cancer (DGC) associated with E-cadherin was observed in 1994, when Becker and colleagues identified somatic E-cadherin mutations in specimens of sporadic DGC [26]. Later on, Guilford P. et al. presented a large kindred from New Zealand with multiple cases of early onset DGC (EODGC) that were carriers of a causative germline mutation in the E-cadherin gene [23]. In the following years, several other publications emerged, confirming the autosomal-dominant pattern of inheritance associated with germline mutations of the *CDH1* gene [24,25,27,28,29,30,31]. Inactivation of the remaining functional allele, by a second hit molecular mechanism, leads to biallelic inactivation of the E-cadherin gene and is the trigger event for the development of diffuse type gastric cancer in germline mutation carriers [8,32,33]. Interestingly, hypermethylation was demonstrated to be the most frequent cause of a second-hit *CDH1* inactivation in HDGC tumours, whereas a second mutation or deletion is apparently less frequent [8,32]. In the sporadic forms of DGC, a hot spot region between exons 7 and 9 is observed for *CDH1* germline mutations, while in the hereditary forms of DGC, the *CDH1* genetic alterations are scattered over the entire gene length [34]. To date, 155 different mutations were identified in members of these families, and no correlation has been reported between the clinical phenotype and the location/type of the mutation presented [25,35]. Furthermore, in about 80% of the cases, *CDH1* germline mutations are of the truncating type, resulting in the complete loss of E-cadherin expression due to the occurrence of premature stop codons [36,37]. However, in about 20% of the HDGC patients, mutations are of the missense type, resulting in full-length E-cadherin molecules with a single amino acid substitution [27,35,36,38]. In the latter, the impact on protein function is not predictable and, for that reason, *CDH1* germline missense mutations represent a serious problem in terms of genetic counselling and clinical surveillance [27,35,36,38].

## 5. Management of *CDH1* Germline Missense Mutation Carriers

**Figure 1:**
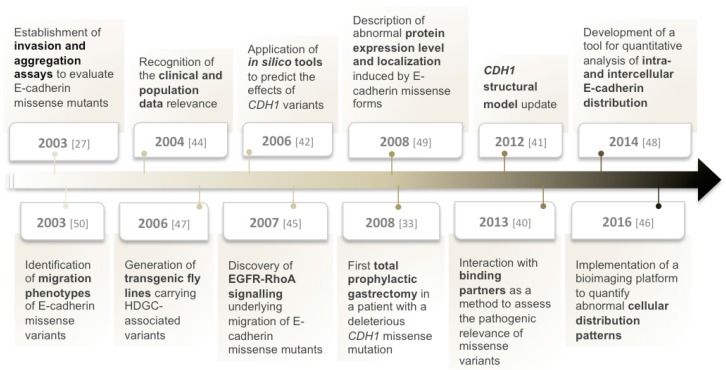
Timeline with the key findings and technological developments important to evaluate the pathogenicity of *CDH1* germline missense variants, in the context of Hereditary Diffuse Gastric Cancer (HDGC) [27,33,40,41,42,44,45,46,47,48,49,50].

The clinical and functional relevance of *CDH1* missense mutations is still controversial, in part because normal protein length and an apparent regular level of expression are observed. Therefore, upon identification of a *CDH1* missense variant, it is mandatory that additional studies are performed to assess how this alteration could perturb the expression and function of E-cadherin, as well as related signaling and cellular mechanisms [25,35,39]. Altogether, those features will determine E-cadherin putative pathogenicity. In clinical terms, this information is extremely valuable, as once a germline missense mutation is detected and classified as deleterious, *CDH1* mutant carriers enter a surveillance programme similar to that offered to carriers of truncating mutations, possibly involving prophylactic surgery [25,35,39]. Thus, in the last decade, and due to the lack of comprehensive tools, Seruca’s group established a multidisciplinary approach to evaluate the pathogenicity of germline *CDH1* missense variants and classify them as neutral or deleterious (pathogenic variants) (Figure 1) [27,38,40,41,42]. The pipeline is extensive and relies on familial data, in silico studies, expression analysis, and functional characterization of *CDH1* missense mutants in vitro and in vivo [27,40,41,42]. Based on the results of these analyses, and upon clinical recommendations, a subset of patients with deleterious germline missense mutations performed prophylactic gastrectomy and the histopathological examination of the stomach revealed the presence of cancer foci in all the specimens, supporting the reliability and the accuracy of this evaluation [33,43]. Regrettably, for about 17% of missense mutations, the current pipeline is not sufficient to ensure a confident result, and the functional relevance of the missense variants remains undetermined. Therefore, carriers of unclassified missense variants should be closely monitored and managed by clinicians, and an intensive endoscopic surveillance programme should be carried out.

## 6. Clinical and Familial Data Collection for Classification of *CDH1* Germline Missense Mutation Carriers

In the last meeting of the International Gastric Cancer Linkage Consortium (IGCLC), the clinical criteria and guidelines for HDGC family screening and surveillance were re-established [25]. In particular, the Consortium proposed that the analysis of genetic and familial data should be the first approach in the evaluation of a *CDH1* missense variant. Moreover, special attention should be given to the presence of gastric and lobular breast cancer (LBC) within the family, as well as the occurrence of cleft/lip congenital malformations [25]. The genealogy allows the analysis of co-segregation of the mutation with the disease within pedigrees and, thus, the identification of inheritance patterns in the family. Fitzgerald and Caldas [44] proposed that, in the case of *CDH1* germline missense variants, at least 4 affected family members need to be screened and present the same alteration. Unfortunately, for most of the families, it is not possible to perform these studies, since geneticists are frequently confronted with families of small size and/or with a low number of affected members within a family, which prevents segregation analysis [42,44]. It is interesting that, and still not yet well understood why, HDGC families with germline *CDH1* missense mutations often display a low disease penetrance [35,42]. Besides the segregation analyses, it is mandatory to evaluate other genetic parameters, such as mutation recurrence in unrelated HDGC families and mutation frequency in healthy controls [25,27,44]. Variant frequency in a control or a general population can be assessed by searching publicly available population databases such as 1000 Genomes Project (http://browser.1000genomes.org), Exome Variant Server (http://evs.gs.washington.edu/EVS), or dbSNP Database (http://www.ncbi.nlm.nih.gov/snp). However, one should be aware that these databases can have limitations as low-quality data and may lack details on the origin of the study or information regarding any possible associated phenotype [51].

## 7. In Silico Predictions of *CDH1* Missense Mutation Pathogenicity 

In silico tools are continuously being developed to improve the knowledge and interpretation of DNA variants. Predictions can provide useful information regarding the effect of the variant on the primary and alternative gene transcripts, as well as the potential impact of the variant on protein structure and function [41,42]. Most of the existing algorithms take into account the degree of conservation of a particular nucleotide among species, the location and context within the protein sequence, the biochemical properties of the amino acid substitution, the putative impact of the variant in protein native-state, and the possible effect in splice sites [41,42]. The use of multiple software programs for sequence variant interpretation is also recommended, as these programs are based on distinct algorithms that result in different outputs [51]. To infer the impact of *CDH1* germline missense mutations using in silico analysis, SIFT and PolyPhen2 have become the standard tools [30,41,42,52,53,54,55,56]. SIFT-Sorting Intolerant From Tolerant (http://sift.jcvi.org/) predicts the impact of a particular amino acid replacement in protein function [57,58]. The method takes into consideration the evolutionary conservation of amino acids within species. Highly conserved residues are expected to be important for protein function, whereas those with a low degree of conservation are likely to tolerate a number of substitutions without affecting the molecule and its cellular function. SIFT workflow ends in a scaled probability, termed the SIFT score, that ranges from 0 to 1 [57]. A substitution is classified as damaging if the score value is below 0.05 [41,42]. It is noteworthy that the software does not use protein structural information, lacking possible compensatory effects of neighbouring positions [59]. In contrast, PolyPhen-2—Polymorphism Phenotyping v2 (http://genetics.bwh.harvard.edu/pph2/) [60,61] employs a machine-learning classification along with a multiple protein sequence alignment pipeline, which combines structural and comparative evolutionary considerations to evaluate effects on protein stability and function [61]. Given that an amino acid replacement in a protein sequence can change many of its chemical and physical properties, resulting in unfolding and decreased stability of polypeptides, structural modelling has become a major tool. Indeed, FoldX (http://foldxsuite.crg.eu/) [62] has been extensively explored to determine the structural impact of *CDH1* missense mutations. Specifically, this theoretical tool calculates how sequence variants, in comparison to the wild-type, affect the native-state stability of the structures (ΔΔG = ΔGMut − ΔGWT), and if the stability change (ΔΔG) is higher than >0.8 kcal/mol, the missense variant is considered destabilizing [62]. Such mutations are associated with high turnover of the protein in the cell, protein premature degradation and, consequently, loss of E-cadherin function [41]. This model covers most of E-cadherin, including the prodomain, the extracellular domain, and the β-catenin binding domain [41]. However, the juxtamembrane region remains to be structurally characterized. 

An additional tool is the Netgene2 algorithm (http://www.cbs.dtu.dk/services/NetGene2/) that investigates the potential of *CDH1* variants to cause alternative splicing and processing of introns in nuclear pre-mRNA [63,64]. Nevertheless, the identification of cryptic splice sites in *CDH1* mutated gene is limited due to the lack of transcript data available. Overall, in silico predictions can be very useful for gathering information, but should be used with caution, as a complementary tool, and not as the sole source of evidence to classify a missense variant [41,42].

## 8. Characterization of *CDH1* Missense Mutations In Vitro

### 8.1. CDH1 Germline Missense Mutation Categorization According to Protein Expression

**Figure 2:**
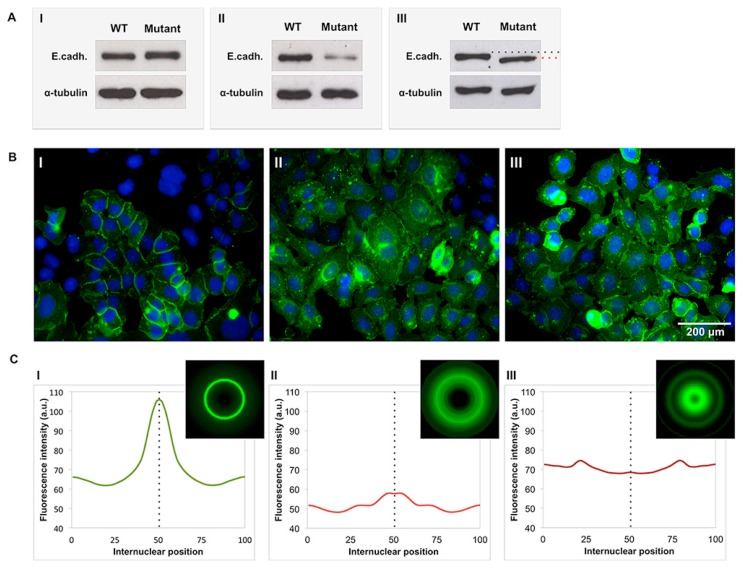
Representative E-cadherin expression profiles in cells with *CDH1* missense variants found in the context of HDGC. (**A**) Chinese Hamster Ovary (CHO) cells transfected with different *CDH1* missense variants were analysed for total E-cadherin expression levels by Western blot. Demonstrative images of normal expression level (**AI**), low expression (**AII**) and abnormal glycosylation (**AIII**) of the protein are shown. Small dots in (**AIII**) represent band mobility shift of total E-cadherin. Tubulin was used as a loading control. (**B**) Immunofluorescence (IF) images (400×) of E-cadherin with a membrane phenotype (**BI**), diffuse subcellular localization (**BII**), and cytoplasmic accumulation (**BIII**). E-cadherin is labelled in green and nuclei are counterstained with DAPI (blue). (**C**) Average intensity of E-cadherin internuclear profiles (IN) and the corresponding virtual illustration, obtained through IF images of cells expressing *CDH1* with distinct missense mutations. Examples for each type of protein accumulation are illustrated. Protein at the membrane, diffused throughout the cytoplasm and perinuclear accumulation of the protein are represented in (**CI**), (**CII**), and (**CIII**), respectively. (a.u.), arbitrary units. The data are in accordance to previously described methods [40,48,49] and emphasize the diversity of *CDH1* missense variants phenotypes.

As previously mentioned, *CDH1* germline variants can result in a normal length protein with localization of the molecule at the membrane. However, missense mutations frequently lead to abnormal E-cadherin levels and expression patterns through mechanisms of trafficking deregulation and premature degradation of the molecule that are most often difficult to detect and interpret [17,49]. Therefore, our group developed a strategy to quantify and map E-cadherin expression for all *CDH1* germline variants by combining Western-blotting, immunocytochemistry, and bioimaging techniques (Figure 2). Briefly, our approach involves the use of an immortalized cell line in which *CDH1* variants are induced. Chinese Hamster Ovary (CHO) cells, which are negative for E-cadherin expression, are transfected with vectors encoding the wild-type E-cadherin or the diverse variants identified at the germline level [27,40,41,42,49,50,52,53,54,55,56,65,66,67]. Upon transfection, protein expression is assessed by Western blot (Figure 2A). Low E-cadherin levels strongly indicate structural destabilization and degradation of the protein by mechanisms of Protein Quality Control (PQC) [41,49]. Occasionally, a band mobility shift can also be detected, indicating that the mutation could affect glycosylation sites [9,68].

Subsequently, immunostaining with monoclonal antibodies is used for E-cadherin analysis at the cellular and intercellular level (Figure 2B). The qualitative evaluation of E-cadherin expression and localization is performed by the classical approach, which involves visual inspection under a fluorescence microscope. Still, this process is strongly operator-dependent and based on subjective criteria. To overcome this limitation, we developed an objective and quantitative methodology that extracts detailed information on E-cadherin distribution intracellularly and at boundaries of contiguous cells (adherens junctions) [48,69]. This tool generates an inter- and intra-cellular expression profile for the wild-type and mutated forms of E-cadherin, and deviations from the reference are used to classify the level of E-cadherin dysfunction (Figure 2C) [48]. Typically, deleterious variants show aberrant peaks of E-cadherin cytoplasmic accumulation, or low and diffuse expression throughout the cell, as a result of trafficking anomalies [17,48,49,69]. For each E-cadherin variant, features such as mean fluorescence intensity at the membrane, position of the maximum fluorescence intensity, and Maximum Mean Ratio (MMR) are computed and subject to analysis. The mean fluorescence intensity, measured at the middle axis between two juxtaposed cells, reflects the number of molecules present at the membrane and is significantly lower in dysfunctional mutants than in wild-type cells [48]. To quantify the variation of the fluorescence signals along the inter-nuclear space, the MMR parameter is used. High MMR values are associated with a strong and well-defined pattern of expression at the membrane, while a low MMR level indicates diffuse protein expression at the membrane and aberrant expression patterns throughout the cell. Accordingly, deleterious variants show a lower MMR when compared to the wild-type form [48]. 

In conclusion, quantification and characterization of E-cadherin expression patterns is crucial to detect deregulated post-translational mechanisms induced by *CDH1* pathogenic mutations.

### 8.2. CDH1 Germline Missense Mutation Classification According to Its Impact on Intercellular Organization and Cell-Cell Adhesion Status

**Figure 3:**
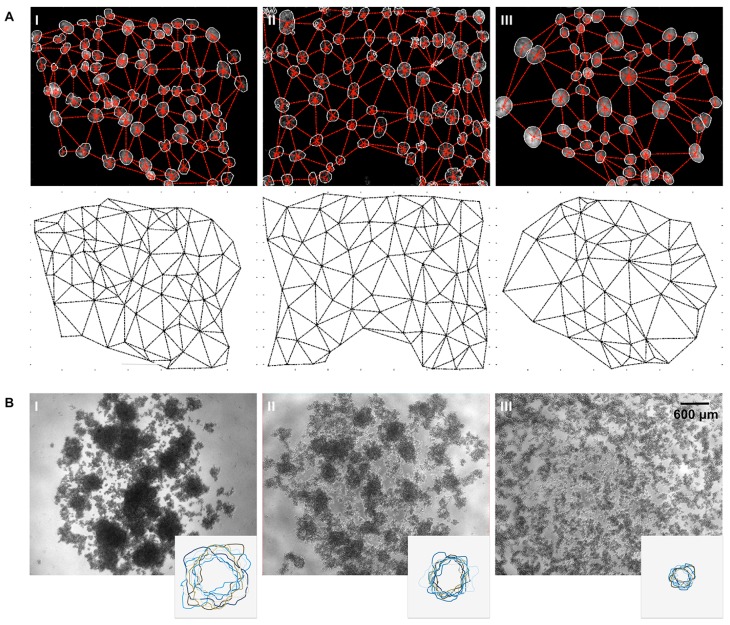
Intercellular organization and cell adhesive properties induced by *CDH1* germline missense variants. (**A**) Patterns of cellular distribution elicited by E-cadherin variants. In the upper panel, cell nuclei are overlapped with the corresponding network. In the lower panel, the final networks are presented. (**AI**) Illustrates a more regular and cohesive cellular topology, while (**AII**) depicts an intermediate cellular organization, and (**AIII**) represents a scatter and disorganized phenotype. (**B**) Adhesiveness of cells expressing E-cadherin variants evaluated by slow-aggregation assays and corresponding outlines of cellular aggregates. Variants preserving a functional adhesion complex display compact cellular aggregates (**BI**), while dysfunctional E-cadherin forms present small cellular aggregates (**BII**) or an isolated phenotype (**BIII**). The images illustrate different adhesiveness effects on E-cadherin missense variants cells as firstly described in [40,46].

Cell adhesion is an essential mechanism in the formation and maintenance of cell architecture in the epithelium [1]. Importantly, functional impairment of E-cadherin and eventual loss of the molecule is typically associated with decreased cell-cell interaction and tissue remodelling [3,5,6]. Recently, to characterize defects in the epithelial structure and morphology that can arise from E-cadherin mutants, we developed a platform that identifies and quantifies cellular distribution patterns using in situ microscopy images [46]. More specifically, we used DAPI-stained nuclei to create artificial cellular networks, from which we could extract quantitative data regarding cell distribution and organization (Figure 3A). The software creates digital meshes composed of triangles centred in triplets of neighbouring nuclei, and explores their topological features, such as area, edges length, and angles [46]. Pathogenic mutations with impact in cellular organization, present triangles with higher areas and edges when compared with the wild-type cell counterparts. At the individual cell level, the assessment of E-cadherin binding with its different cytoplasmic protein partners is important as part of the missense mutation studies [40]. Notably, the cytoplasmic domain of E-cadherin has a crucial role in its function, because it supports the assembly of a complex of cytosolic proteins, including α-, β-, p120-, and γ-catenins, which provides anchorage to the actin cytoskeleton to form stable cell-cell contact [2,3]. Nonetheless, this domain also has an essential role in protein trafficking and regulation at the membrane [14,70]. The association of β-catenin and PIPKIγ to E-cadherin cytoplasmic portion is necessary for newly synthesized E-cadherin molecules to be delivered to the basolateral membrane [15,71]. For maintenance and stability of the molecules at the membrane, p120-ctn binds to the juxtamembrane domain of E-cadherin and simultaneously blocks the interaction with the endocytic machinery, such as with clathrin adaptor proteins and Hakai [19,72,73]. Importantly, Hakai binds directly to E-cadherin and, being an E3 ubiquitin-ligase, it ubiquitinates and induces E-cadherin endocytosis [19]. To verify the interaction of E-cadherin with its various interactors, we use an indirect approach, the in situ Proximity ligation assay (PLA) [40]. This assay, which is a PCR-based system, relies on the affinity between two proteins requiring their proximal binding to get an amplification signal that can be detected at the cellular level [74,75]. Therefore, we have used in situ PLA to determine which *CDH1* missense variants, located at the cytoplasmic domain of the protein, affect the correct interplay with the corresponding binding partners [40]. Using this strategy, we have identified E-cadherin mutations that impair the association of E-cadherin/β-catenin, some located outside of the E-cadherin β-catenin binding domain [40]. Further, we have also demonstrated that E-cadherin mutations affecting the p120-binding domain are more available to be targeted by Hakai and to be degraded, and in this way to behave functionally as a truncated mutation [40]. Interestingly, the PLA results point out that each mutation behaviour is unique, as it interacts differently with its binding partners, produces its own phenotype, and possibly plays different roles in signal transduction. For these reasons, we believe that each E-cadherin missense mutation is likely to induce cell-specific biological behaviour.

Furthermore, due to the pivotal role of E-cadherin in cell-cell adhesion, understanding how E-cadherin impacts this cellular effect is of major relevance. Indeed, we have established a functional in vitro cell model to determine the impact of *CDH1* variants on cell compaction, a direct indicator of cell-cell adhesion competence [27,42,65]. In this assay, a single-cell suspension is seeded on soft-agar, and cells with a competent adhesion complex spontaneously aggregate (Figure 3B). Accordingly, cells transfected with the wild-type protein form compact cellular aggregates, while cells expressing dysfunctional E-cadherin form small cellular aggregates with different degrees of cohesion, or a completely isolated phenotype. The areas and density of the aggregates are subsequently quantified for a complete evaluation of cellular adhesiveness. Overall, using these different approaches, we established a system for a thorough characterization of *CDH1* variants with respect to their effect on cellular topology, stability of the cadherin-catenin complex, and strength of cell-cell interactions.

### 8.3. Invasive and Migratory Properties of Cells with CDH1 Germline Missense Mutations

Gastric cancer of the diffuse type is a highly invasive and lethal cancer, as cells that lose E-cadherin can evade apoptosis stimuli and acquire increased cell invasive potential, determining the fast and silent progression of the disease [34,36,76]. Therefore, assessing the ability of directed migration and spread throughout the extracellular matrix is of major importance in the study of E-cadherin missense variants [45,50,77,78,79]. 

**Figure 4:**
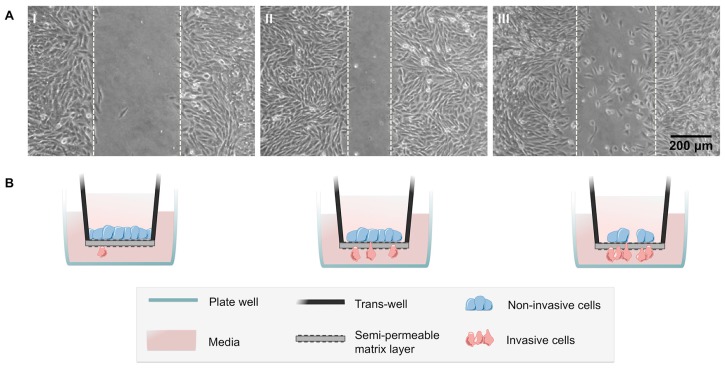

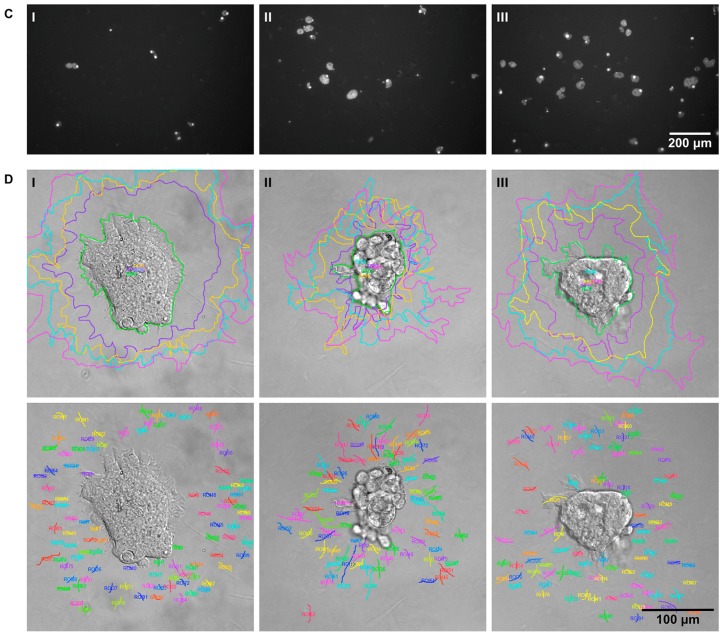
Effect of *CDH1* germline missense variants on the invasive and migratory properties of cells. (**A**) Migratory behaviour of Chinese Hamster Ovary (CHO) cells transfected with *CDH1* variants. Panel (**AI**) illustrates cells with decreased motility, (**AII**) exhibits a compact front of migration with unidirectional movement of cells, and (**AIII**) shows random colonization of the wound. Pictures were captured in phase contrast microscopy (200×), 8 h after wound incision. (**B**) Illustrative scheme of the non-invasive, invasive, and highly invasive phenotypes. (**C**) Invasive behaviour of cells expressing *CDH1* variants in matrigel-coated insert wells. The invasive cells on the lower part of the insert membranes were stained with DAPI. (**CI**) Represents cells with a non-invasive phenotype, (**CII**) represents cells with an invasive phenotype, and (**CIII**) shows cells with a high invasion rate. (**D**) Structural organization (upper panel) and protrusion formation (lower panel) of cellular spheroids embedded in collagen were monitored by time-lapse (400×). The area, as well as protrusion trajectories over time, are marked by colored traces. Cells forming compact aggregates and short protrusions are displayed in (**DI**). (**DII**) Shows a small multicellular structure with lower number of cells but more extended protrusions. In (**DIII**), cells form a more extensive and disorganized structure with large protrusions, indicating a highly invasive phenotype. The data in panels (**A**–**C**) are in accordance with previously described methods [27,50,79]. Novel strategies and phenotypes to better evaluate *CDH1* mutation variants are illustrated in panel (**D**).

A series of methodologies is used to evaluate the invasive and migratory properties of cells with *CDH1* germline missense mutations. Most frequently, the motile/migratory behaviour of the cells, transfected with the wild-type protein or the missense variants, is evaluated using wound reepithelialisation systems (Figure 4A). The method requires unilateral adhesion and transient attachment to a substrate, usually fibronectin, and provides information regarding migration velocity, persistence, and directionality during wound healing [80,81]. By exploiting this approach, we have identified a subset of germline E-cadherin missense variants that are associated with particular cellular phenotypes and biological behaviours [50]. Indeed, missense mutations clustering in the extracellular region of E-cadherin lead to cytoskeleton rearrangements and fibroblastic morphology, which provide cells with increased motility [50]. Cells expressing those variants migrate in an isolated and random way, and faster than the wild-type cells or the cells with variants affecting the intracellular portion of E-cadherin [50]. Further, we verified that E-cadherin-dependent migration is mediated by reduced E-cadherin/EGFR interaction and, consequently, by aberrant activation of EGFR and RhoA-GTP [45,79]. In contrast, intracellular mutants, such as V832M, display piled-up structures of round cells and migrate collectively and in a directed manner across the wound due to a reduced affinity between β-catenin and α-catenin [50]. Alternatively, the effects of the *CDH1* deleterious variants in cell motility can also be analysed independently of a wound stimulus by time-lapse scanning microscopy. This assay, although corroborating the wound healing data, is only used as a complementary tool [79]. To evaluate the invasive ability of E-cadherin mutant cells, matrigel invasion chambers are the in vitro preferred system [27,40,41,42,49,53,54,55,56,65,66]. The matrigel matrix contains structural proteins such as collagen, fibronectin, laminin, and proteoglycans, but also a panel of growth factors, which reconstitute the basement membrane composition and provide proper conditions for cell interaction with the surrounding microenvironment [82,83]. Upon seeding, invasive cells are able to degrade the matrix and reach the lower side of the filter through the pores (Figure 4B,C). The total number of invasive cells is then counted using a fluorescent microscope. In contrast, non-invasive cells do not migrate through the membrane and remain in an epithelium-like structure on top of the matrigel (Figure 4B). Remarkably, about 60% of the variants studied to date were shown to be invasive [27,40,41,42,49,53,54,55,56,65,66].

As the process through which invasive cells leave the epithelium and cross the basement membrane involves proteolytic degradation by the matrix metalloproteinase (MMPs) [84], additional assays can be performed to evaluate whether cells harbouring deleterious variants of E-cadherin lead to increased protease secretion. Although informative, this assay is not routinely used. Very recently, and taking advantage of the morphological changes that cells undergo to escape the epithelium and invade adjacent tissues, we have established an innovative approach using 3D culture systems. In order to analyse structural organization, protrusion formation, and dissemination of cells with E-cadherin variants, spheroids of wild-type and mutant cells are embedded in collagen and monitored by time-lapse. The aggregate area, the number of cells that disseminate, as well as the number and extension of protrusive structures can be easily evaluated (Figure 4D). In line with this, our next step is to track different cytoskeletal markers in these cells, and to develop new algorithms and software applications to analyse their patterns and dynamics. We envision that our combined strategy will be able to identify deleterious E-cadherin variants more efficiently and provide novel insights into the clinical surveillance of *CDH1* mutation carriers.

## 9. Assessment of *CDH1* Germline Missense Mutation Aggressiveness through an In Vivo Strategy

**Figure 5:**
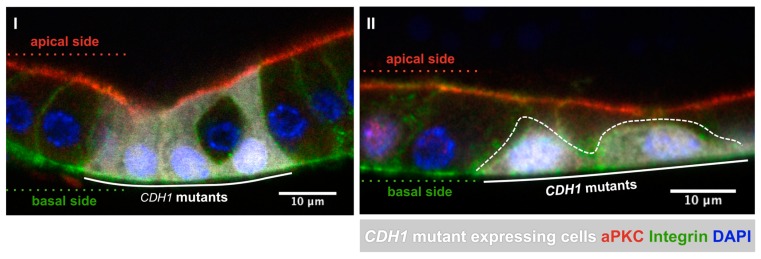
Expression of human *CDH1* mutants in the *Drosophila* follicular epithelium and their effects on tissue organization. (**I**) The expression of *CDH1* mutants promotes the disruption of epithelial organization, inducing epithelial invagination; (**II**) Cell extrusion from the monolayer occurs through loss of contact with the apical surface of the tissue. Staining is as follows: DAPI labels the nuclei, aPKC in red delineates the apical side, whereas integrins are labelled in green. Mosaic expression of *CDH1* (labelled in white) enables direct comparison between wild-type and genetically manipulated clones. The original data in *Drosophila* heighten the potential of novel strategies to evaluate *CDH1* missense variants in the context of HDGC.

In addition to in vitro studies, the use of animal models is of major relevance to better understand the molecular mechanisms of cancer development. While mice models are frequently used in in vivo studies, there are many limitations associated with this model in the context of gastric cancer [85,86,87]. Therefore, the use of alternative organisms has been suggested to study specific features of cancer development. In particular, *Drosophila melanogaster* has received much attention as it is an inexpensive, genetically tractable organism that can recapitulate key events of human carcinogenesis, allowing investigation of cell morphology, invasion, and metastatic growth. Moreover, there is a high degree of conservation in terms of the basic mechanisms and signaling pathways in flies and man. Junctional complexes and overall epithelial organization are similar enough, in vertebrates and invertebrates, to assume that most cellular and molecular mechanisms involved in epithelial maintenance and reorganization are conserved [88]. Taking into account these similarities, the *Drosophila’s* potential has been explored to unravel the cascade of events that follow E-cadherin loss of function due to missense mutations and to understand how they contribute to cancer progression in the tissue, in an in vivo context. Suriano G. and colleagues [47] generated transgenic fly lines carrying cDNAs of wild-type human E-cadherin (hEcad) and two missense mutant forms obtained from HDGC patients: hEcad-A634V, which affects the extracellular protein domain, and hEcad-V832M, affecting the intracellular portion. Using a GAL4/UAS system, the different hE-cadherin forms were expressed in the *Drosophila*-developing wing epithelium (the so-called wing imaginal disc) that forms a simple monolayer epithelium and allows the inspection of an altered pattern of E-cadherin sub-cellular localization [47]. Interestingly, it was observed that cells expressing the wild-type protein remain confined to normal epithelial fold as a result of proper cell-cell interaction [47]. In contrast, the mutant cells expressing A634V and V832M forms were found to infiltrate neighbouring regions of wing epithelium [47]. Remarkably, the mutants exhibited unlike behaviours regarding its invasive pattern, possibly due to distinctive abilities to support cell-cell adhesion. The A634V mutant still retains homophilic adhesion, invading as a group of cells, whereas the hE-cadherin V832M has a stronger effect on the adhesive capabilities and invades as smaller groups of cells or even individually [47]. Furthermore, it was shown that the fly β-catenin homolog, Armadillo (Arm), could mediate the distinct migratory and invasive behaviours of the different E-cadherin forms. In accordance, overexpression of hE-cadherin V832M in *Drosophila* imaginal disc exposed a weaker interaction with Arm at the plasma membrane and, thus, the availability of Arm for the canonical Wtn-Notch signaling activation [47]. Those results recapitulated the in vitro findings for both mutations [50], validating the applicability of the in vivo assays in the characterization of HDGC-associated germline missense mutations. More recently, we are using the *Drosophila* ovary as a model to evaluate novel HDGC-associated *CDH1* variants and their impact on epithelial organization and cell migration (data not shown). More specifically, we are able to easily analyse the influence of *CDH1* variants on the monolayered follicular epithelium (Figure 5) and also the effects of specific human cadherin transgenes on the collective migration of epithelial cysts formed by border cells. To date, all the mutants studied affected E-cadherin expression at the membrane and frequently disrupted epithelial organization, mimicking what is observed in biological samples from HDGC patients [35,36]. Additionally, to investigate migration dynamics of cells carrying E-cadherin variants in vivo, the fly dorsal closure model is also being tested. Particularly, using live imaging and fluorescently tagged transgenes, we are able to monitor closure rates, zippering velocity, and epithelial cohesion, as well as leading edge morphology and orientation in opposing migratory epidermis towards the dorsal midline of the embryos [89,90]. In conclusion, the use of the *Drosophila* model could have a huge impact in the current pipeline for the characterization of *CDH1* variants, as well as for research purposes in the context of targeting interactors and signaling pathways mediated by E-cadherin dysfunction.

## 10. Conclusions

Alterations in *CDH1*/E-cadherin are the proven cause for HDGC and LBC [34,36,76]. In these cancers, loss of E-cadherin function alters cell morphology and epithelial architecture, disrupts cell-cell adhesion, and increases cancer invasion, contributing to the high mortality rate of gastric cancer [3,4]. In case a germline pathogenic mutation is identified, the carrier is counselled to perform the ablation of the target organ, since the disease is silent and has a very poor prognosis. The clinical guidelines for truncating mutations are well established, but *CDH1* missense sequence variants still pose a clinical burden for geneticists and clinicians [27,35,36]. Therefore, and in the absence of appropriate clinical and familial data, the characterization of these *CDH1* variants and their classification, as neutral or deleterious, is mandatory.

As a reference centre of the IGCLC, our group has established a series of functional assays and developed novel approaches to assess the pathogenic role of all *CDH1* germline sequence variants detected worldwide (from New Zealand, Europe, and Asia to North and South America) and show their added value in genetic counselling [27,40,42,46,48]. In close collaboration with experimental biologists, bioinformaticians, and bioengineers, we show herein how these methods contribute to ameliorating the classification of E-cadherin germline mutations. Based on our multidisciplinary approach, curative prophylactic gastrectomy has already been performed in carriers of germline missense mutations and histopathological examination of the gastrectomies revealed the presence of invasive cancer in all the specimens, supporting the reliability of this working model [33,43]. We envisage that, in the near future, the development of novel and user-friendly tools will further improve the identification and management of deleterious variant carriers reported in genetic screening.

## Abbreviations

ARF6	ADP-ribosylation factor 6
Arm	Armadillo
CHO	Chinese Hamster Ovary
DGC	Diffuse Gastric Cancer
DAPI	4′,6-diamidino-2-phenylindole
EGFR	Epithelial growth factor receptor
EODGC	Early onset Diffuse Gastric Cancer
ERAD	Endoplasmic reticulum-associated degradation
HDGC	Hereditary Diffuse Gastric Cancer
hEcad	Human E-cadherin
IGCLC	International Gastric Cancer Linkage Consortium
LBC	Lobular Breast Cancer
miRNAs	MicroRNAs
MMPs	Matrix metalloproteinase
MMR	Maximum Mean Ratio
PIPKIγ	Type Iγ phosphatidylinositol phosphate Kinase
PLA	Proximity ligation assay
PolyPhen-2	Polymorphism Phenotyping v2
PQC	Protein Quality Control
SIFT	Sorting Intolerant From Tolerant
ZEB	Zinc finger E-box-binding homeobox
